# Autoimmune Diseases and Oral Health: 30-Year Follow-Up of a Swedish Cohort

**DOI:** 10.3390/dj6010001

**Published:** 2017-12-22

**Authors:** Anna Julkunen, Anna Maria Heikkinen, Birgitta Söder, Per-Östen Söder, Sanna Toppila-Salmi, Jukka H. Meurman

**Affiliations:** 1 Department of Oral and Maxillofacial Diseases, University of Helsinki and Helsinki University Hospital, 00014 Helsinki, Finland; anna.julkunen@helsinki.fi (A.J.); anna.m.heikkinen@helsinki.fi (A.M.H.); sanna.salmi@helsinki.fi (S.T.-S.); jukka.meurman@helsinki.fi (J.H.M.); 2 Department of Dental Medicine, Karolinska Institutet, BOX 4064, 14104 Huddinge, Stockholm, Sweden; perosten.soder@telia.com

**Keywords:** autoimmune disease, oral health, association, plaque index, follow-up study

## Abstract

Oral infections up-regulate a number of systemic inflammatory reactions that, in turn, play a role in the development of systemic diseases. We investigated the association between oral health and autoimmune diseases in a cohort of Swedish adults. Hypothesis was that poor oral health associates with incidence of autoimmune diseases. Overall 1676 subjects aged 30–40 years old from Stockholm County (Sweden) participated in this study in 1985. Subjects were randomly selected from the registry file of Stockholm region and were followed-up for 30 years. Their hospital and open health care admissions (World Health Organization ICD 9 and 10 codes) were recorded from the Swedish national health registers. The association between the diagnosed autoimmune disease and the oral health variables were statistically analyzed. In all, 50 patients with autoimmune diagnoses were detected from the data. Plaque index was significantly higher in the autoimmune disease group (≥median 35 (70%) vs. ˂median 872 (54%), *p* = 0.030). No statistical difference was found in gingival index, calculus index, missing teeth, periodontal pockets, smoking or snuff use between patients with and without autoimmune disease. Our study hypothesis was partly confirmed. The result showed that subjects with a higher plaque index, marker of poor oral hygiene, were more likely to develop autoimmune diseases in 30 years.

## 1. Introduction 

Autoimmune diseases are rare pathological states arising from an abnormal immune response to substances and tissues that are normally present in the body. These diseases are multifactorial, heterogeneous and variable conditions that may exist in several organs and cell types [1,2]. The pathomechanisms of autoimmunity are multifactorial and mostly unknown [1]. The stability and functionality of tissues is a complex and strictly regulated process where immune system plays a role [2]. Pathogens can affect the regulation and autoimmunity reactions may follow [2]. Infection can induce autoimmunity either via the innate or adaptive immune responses [3].

A strong link has indeed been shown between viral, bacterial and other microbial infections and autoimmunity [3,4]. However, there are many factors that affect autoimmune diseases, like genetics, age, gender, reproductive status, and hormones [2,5]. Smoking tobacco also associates with autoimmune diseases but this has not been observed regarding snuff use [6,7]. 

From a clinical perspective there are two ways to categorize autoimmune diseases; organ-specific or systemic [8]. In organ-specific autoimmune diseases the expression of autoimmunity is limited to specific organs, for example on insulin-producing β-cells in pancreas in Type-1 diabetes mellitus [8,9]. In systemic autoimmune diseases autoimmunity affects multiple organs, such as seen in rheumatoid arthritis (RA) [10]. Some of the immune-mediated diseases also affect oral mucosa [11]. These findings can in fact be the first signs of manifestation of an autoimmune disease [11]. Oral infections as such up-regulate a number of systemic inflammatory reactions which, in turn, play a role in the development of many systemic diseases [11].

The American Autoimmune Related Diseases Association (AARDA) identifies 80–100 different autoimmune diseases and at least 40 additional diseases have been suspected to have autoimmune characteristics [12]. Autoimmune diseases are chronic, often progressing and severe [12]. 

Periodontitis is a multifactorial inflammatory disease where tissues around the teeth can destruct and finally leading to loss of teeth [13]. Periodontal pocket depth ≥5 mm is considered as severe clinical attachment loss [14]. Periodontitis is associated with a number of diseases like the above-mentioned RA and diabetes [15,16]. Since 1985 we have investigated systematically the associations between oral infections, periodontitis in particular, with several general diseases in a Swedish population cohort. Our studies have shown statistically significant associations between poor oral health and cardiovascular diseases and cancer [17,18,19]. In the same cohort we now investigated the incidence of autoimmune diseases with respect to the patients’ oral health parameters. The aim of this study was to examine if poor oral health indeed associates with autoimmune diseases, in the infection or inflammation paradigm. The hypothesis was that poor oral health reflects in the incidence of autoimmune diseases.

## 2. Materials and Methods

At baseline in 1985, a cohort of 3273 subjects was randomly selected from the registry file of all inhabitants in the Stockholm region who were born on the 20th of any month from 1945 to 1954. Of them 1676 individuals replied to the invitation and participated in the baseline study. They underwent a clinical oral examination and filled out a structured questionnaire. Basic characteristics and health behavioral such as smoking and snuff use habits and working status were registered (Table 1). The clinical examination included measures for plaque index (PI) [20], gingival index (GI) [20,21], calculus index (CI), recording periodontal pockets and missing teeth (Table 2). CI was scored from 0 (no calculus) to 3 (abundant calculus) according to Greene and Vermillion [22]. Depth of periodontal pockets was measured from all teeth with a Hu-Friedy (PCPUNC 15) periodontal probe (Hu-friedy, Chicago, IL, USA) and pockets ≥5 mm were registered. The number of teeth was also registered. Periodontal pockets and missing teeth (both categorized as yes/no) were used in the analyses. Details of the baseline study have been earlier published [23,24]. There were no patients with dentures in the present data.

In the present study we used a modified list of autoimmune diseases published by AARDA [12]. Only diseases with literature evidence of autoimmunity as main etiology were accepted in the list (Table 3). 

## 3. Ethical Considerations 

The study was approved by the Ethics Committee of the Karolinska Institutet and Huddinge University Hospital in Sweden (Dnr 101/85 and revised in 2012/590-32). The study is in accordance with the Declaration of Helsinki.

## 4. Autoimmune Related Diseases and Socioeconomic Data

Data about autoimmune-related diseases were obtained from the Centre of Epidemiology, Swedish National Board of Health and Welfare, Sweden. The data were classified according to the World Health Organization International Statistical Classification of Diseases and Related Health Problems (ICD-9 and ICD-10). Socioeconomic data were further obtained from the National Statistics Centre, Örebro, Sweden, based on the 1985 file. The cumulated data for the disease incidence from 1985 to 2015 were statistically analysed with the clinical data from 1985.

## 5. Data Analysis

Statistical analyses were carried out by the SPSS Base 15.0 Statistical Software Package (SPSS Inc., Chicago, IL, USA). Comparisons were made by cross-tabulation, chi-square test, and binary logistic regression. Median values of PI, GI and CI were calculated. We analyzed the association between patients with and without autoimmune disease and the following variables: sex, smoking (current smoker/ex-smoker/non-smoker), snuff use (current snuffer/ex-snuffer/non-snuffer), working status (yes/no currently working), ≥5 mm periodontal pockets (yes/no), missing teeth (yes/no) and median values of PI, GI, and CI scores, respectively. *p*-values less than 0.05 were considered statistically significant.

## 6. Results

Patient characteristics are given in Table 1. The autoimmune diagnoses found among the patients are given in Table 3. The subjects were separated into two groups: patients with (N = 50) and with no (N = 1626) autoimmune disease (Table 2). Their oral health and background variables were statistically analyzed and compared to each other. The gender distribution was the same in both groups. Smoking and snuff use did not differ significantly between the groups. However, a trend was found according to patients with autoimmune disease being on average more seldom in working life than those with no autoimmune disease diagnosis. 

Oral health data showed no difference between groups in the number of periodontal pockets or missing teeth. The autoimmune patients with ≥5 mm periodontal pockets were diagnosed with diabetes mellitus Type-1 (three patients), rheumatic diseases (4 patients), Henoch-Schönlein purpura (one patient), and colitis ulcerosa (one patient). Twenty-three patients (46%) with autoimmune disease and 720 patients (44%) without autoimmune disease had missing teeth (*p* = 0.885). The number of missing teeth per person varied: 1–6 among the autoimmune disease patients, 1–28 among patients with no autoimmune disease, respectively. 

The presence of autoimmune disease associated with higher PI (crude odds ratio (OR) = 2.00, 95% confidence interval (CI) = 1.09–3.70, *p* = 0.016). When adjusted by gender and use of snuff the result remained the same (adjusted OR = 2.30, 95% CI = 1.17–4.56, *p* = 0.016). No statistical significant difference was found in GI scores of patients with and without autoimmune disease diagnosis. CI scores did not either differ between the groups (Table 2).

## 7. Discussion 

This study was made to investigate if oral health parameters, with emphasis in periodontitis, associate with the presence of autoimmune disease as we had hypothesized. The main finding was that only PI was significantly higher among the autoimmune disease group compared with those without autoimmune disease. High PI reflects poor oral hygiene and may, thus, cause upregulating of cytokines and inflammatory mediators in the tooth supporting tissues. However, the gingival index was no higher in the autoimmune disease group which finding, in this perspective, was surprising. One would expect that the accumulation of dental plaque links to gingival inflammation, too, but this was not the case in the present material. Here the patients’ medication may also have an effect because anti-inflammatory drugs were frequently used by these patients. Data on medication, however, were not in our disposal, which is a limitation of our study.

The role of infections in the etiology and development of autoimmune diseases is not clear [25]. There have been numerous theories how infections could cause autoimmunity for example by molecular mimicry [25]. In the 1950s a theory was presented that self-reactivity (as in autoimmunity) was some kind of failure of the immune system but today it is known that these are normal reactions in regeneration and healing processes [25]. For example dead or dysfunctional cells must be eliminated and removed [25]. Autoantibodies also have a significant role in infections, especially in viral infections, which have been connected to the development of autoimmune diseases [25].

In our cohort, most of the autoimmune diseases were rheumatic disease or Type-1 diabetes. It is known that periodontal disease associates with RA and diabetes, so this finding was not new [26,27,28]. Patients with rheumatic diseases may have problems with manual dexterity and consequent difficulties in cleaning the teeth. If the rheumatic disease has been present already in 1985 at the baseline of this study, or before, perhaps this might be one factor explaining why the patients with autoimmune diseases as a group had higher PI scores compared with the healthy ones. 

To the best of our knowledge the present investigation is the first study to evaluate oral health and the incidence of all autoimmune diseases in the same study ethnically homogenous population. At the time when this cohort study was commenced the Swedish population was mainly homogenous Caucasian, which is a strength even though the results might not be generalized to other populations. 

The limitations of our study, however, are the lack of data of health habits, such as alcohol use and tooth brushing frequency, which had not been recorded. The number of subjects with autoimmune disease was small even though originally more than 3000 subjects had been enrolled to the study; thus, the statistical power remained weak. The fact that the exact time when the diagnosis was made was not included in the register files also was a weakness. Likewise, it was not possible to assess the patients’ liability, such as having close relatives with autoimmunity. Furthermore, the exact reason for teeth loss remains unclear. As well as the relationship of bacterial infection and systemic disease could only be speculated without supporting inflammatory data. 

## 8. Conclusions

The subjects with a higher plaque index appeared to be more likely to develop autoimmune diseases in 30 years. Furthermore, patients with autoimmune diseases also were less frequently in working life probably due to their disease.

## Figures and Tables

**Table 1:** Characteristics and health habits of patients without and with autoimmune disease.

	Patients with No Autoimmune Disease	Patients with Autoimmune Disease	
Number of Patients	N = 1626 (%)	N = 50 (%)	*p*-Value ^a^
**Sex**			0.886
Male	812 (50)	26 (52)	
Female	814 (50)	24 (48)	
**Smoking**			0.710
Current smoker	595 (36.5)	21 (42)	
Former smoker	432 (26.5)	13 (26)	
Non-smoker	599 (37)	16 (32)	
**Snuff use**	^b^	^c^	0.289
Current snuffer	89 (5)	5 (10)	
Former snuffer	28 (2)	0 (0)	
Non-snuffer	1367 (84)	37 (74)	
**Working**			0.073
Currently	1488 (92)	42 (84)	
Not currently	138 (8)	8 (16)	

^a^ *p*-value by Fisher’s exact test; ^b^ No data of 142 patients; ^c^ No data of eight patients.

**Table 2:** Oral health status of the patients without and with autoimmune disease.

	Patients with No Autoimmune Disease N = 1626 (%)	Patients with Autoimmune Disease N = 50 (%)	*p*-Value ^a^
Periodontal pockets ≥5 mm			0.849
Yes	277 (17)	9 (18)	
No	1349 (83)	41 (82)	
Missing teeth			0.885
Yes	720 (44)	23 (46)	
No	906 (56)	27 (54)	
Plaque index (median 0.67)	^b^		0.030
0.00–0.66	749 (46)	15 (30)	
0.67–3.00	872 (54)	35 (70)	
Gingival index (median 1.19)			0.668
0.00–1.18	804 (49)	23 (46)	
1.19–3.00	822 (51)	27 (54)	
Calculus index (median 0.17)	^c^		0.065
0–0.16	538 (33)	10 (20)	
0.17–3.00	1083 (67)	40 (80)	

^a^ *p*-value by Fisher’s exact test; ^b^ No data of 5 patients; ^c^ No data of 5 patients.

**Table 3:** Autoimmune diseases in the material.

Autoimmune Disease	ICD 10	Number of Patients
Ankylosing spondylitis ^1^	M45	1
Crohn’s disease ^2^	K50	6
Colitis ulcerosa ^3^	K51	5
Diabetes mellitus Type-1 ^4^	E10	14
Graves’ disease ^5^	E05.0	1
Guillain-Barré syndrome ^6^	G61.0	2
Henoch-Schönlein purpura ^7^	D69.0	1
Lichen planus ^8^	L43	1
Psoriasis ^9^	L40	5
Rheumatic disease ^10^	M05	15
Sicca syndrome (e.g., Sjögren) ^11^	M35.0	1
Systemic lupus erythematosus ^12^	M32	1
Wegener’s granulomatosis ^13^	M31.3	1

^1^ includes: ankylosing spondylitis (ICD-9 720); ^2^ includes: Crohn’s disease of large intestine without complications (K50.1), Crohn’s disease, unspecified, without complications (K50.9); ^3^ includes: Ulcerative colitis, unspecified (K51.9), Ulcerative (chronic) rectosigmoiditis (K51.3); ^4^ includes: Type 1 diabetes mellitus (E10.0), Type 1 diabetes mellitus without complications (E10.9); ^5^ Includes: Graves’ disease; ^6^ includes: Guillain-Barre syndrome (G61.0); ^7^ Includes: Henoch-Schönlein purpura (ICD9 287); ^8^ includes: other lichen planus (L43.8); ^9^ includes: Psoriasis (L40.0), psoriasis, unspecified (L40.9); ^10^ includes: crystal arthropathy (M11.9), lethal midline granuloma (ICD9 446.3), myalgia (M79.1), other rheumatoid arthritis with rheumatoid factor of multiple site (M05.8), other seropositive rheumatoid arthritis (M05.8), primary osteoarthritis of other joints (M19.0), rheumatic fever without mention of heart involvement (I00.9), seropositive rheumatoid arthritis, unspecified (M05.9), unilateral primary osteoarthritis of hip (M16.1), unilateral primary osteoarthritis of knee (M17.1); ^11^ Sicca syndrome (M35.0); ^12^ includes: systemic lupus erythematosus, unspecified (M32.9); ^13^ includes: Wegener’s granulomatosis (M31.3).

## Abbreviations

AARDA	The American Autoimmune Related Diseases Association
CI	Calculus index
GI	Gingival index
PI	Plaque index
RA	Rheumatoid arthritis
